# FilmQADose: Open‐source software for radiochromic film dosimetry

**DOI:** 10.1002/acm2.70602

**Published:** 2026-05-06

**Authors:** Eduardo Herrera‐Alba, Carlos Contreras‐Quiroz, Juliana Sandoval‐Navia, Jaider Vásquez, Carlos Ávila

**Affiliations:** ^1^ Physics Department Universidad de los Andes Bogotá Colombia; ^2^ Centro de Control de Cáncer Bogotá Colombia

**Keywords:** gamma analysis, open‐source software, quality assurance, radiochromic film dosimetry, radiotherapy

## Abstract

**Purpose:**

*FilmQADose* is an open‐source, Python‐based software for radiochromic film dosimetry, designed to provide medical physicists with a freely available, extensible tool for two‐dimensional dose verification in radiotherapy. It covers the full film QA workflow, from calibration curve generation to plan‐to‐film comparison, targeting clinical and research centers that require an accessible alternative to commercial solutions.

**Development and Validation Methods:**

The software was developed in Python using open‐source scientific libraries for image processing, curve fitting, DICOM handling, and gamma analysis. Core functionalities include rational and polynomial calibration models, multichannel dose reconstruction, normalized cross‐correlation template matching for automatic alignment, and gamma analysis. Validation was performed using irradiated Gafchromic EBT3 films in square field, pyramidal, and breast IMRT plans. Measured dose maps were compared against treatment planning system calculations using 3%/3 mm gamma criteria.

**Data Format and Usage Notes:**

*FilmQADose* processes TIFF images acquired from flatbed scanners and DICOM RT Dose files exported from treatment planning systems. Output dose maps are generated in DICOM 2D format. The software is implemented in Python with standard scientific Python dependencies and includes a graphical user interface built using PySide6. The source code, documentation, and usage guides are publicly available at: eduardoh27.github.io/FilmQADose.

**Potential Applications:**

The software supports clinical quality assurance in IMRT dose verification, as well as for research applications in radiochromic film dosimetry methodology. A limitation is the absence of lateral response artifact correction and recent machine‐learning‐based calibration methods.

## 1 INTRODUCTION

Radiotherapy (RT) treatments have evolved to improve precision and accuracy of dose delivered to target volumes, decreasing or limiting healthy tissue toxicity with the least possible impact on the patient's health. The increasing complexity of planning techniques, implemented using treatment planning systems (TPS), and the actual dependence of dose delivery on multiple mechanical and dosimetric variables make it necessary to ensure that planned doses are delivered precisely and accurately to the patient, performing dosimetric measurements with ionization chamber arrays, semiconductor‐based detectors or gel dosimetry, among others. Techniques such as intensity‐modulated radiation therapy (IMRT) and stereotactic radiosurgery (SRS) still represent a considerable dosimetric challenge due to the presence of high dose gradients, which are not well reproduced or measured by all dosimetry devices.^1^ Radiochromic film dosimetry (RFD) has stood out as one of the best quality assurance (QA) alternatives in two‐dimensional (2D) dosimetry for IMRT, SRS and brachytherapy treatments,^2^, ^3^ due to film's desirable properties such as relative independence on energy,^4^, ^5^ high sensitivity in a wide dose range,^6^, ^7^ high spatial resolution, lack of physical or chemical processing, relatively low cost and near tissue equivalence.^8^

There are various radiochromic film models and manufacturers aimed to cover a wide range of possible applications, from industrial to medical dosimetry in different beam energy and absorbed dose ranges. They differ in the specific composition of the active layer(s), as well as in the structure and properties of coatings protecting them from mechanical stress, oxidation upon contact with air, and residual UV radiation.^9^ Particularly, GAF/ISP/Ashland's,^10^ Gafchromic EBT3,^11^ and EBT‐XD^12^ models are the most widely used for radiotherapy verification purposes, covering dose ranges from 0.01 to 40 Gy. Furthermore, the EBT‐XD model is preferred when the absorbed dose is greater than 10 Gy, while Gafchromic XR line is designed for diagnostic radiology applications.

Radiochromic films act as relative dosimeters,^13^ requiring a calibration procedure to convert the film response into two‐dimensional dose maps. The absorbed dose induces a change in optical density (OD), which is quantified using optical devices such as flatbed scanners^8^, ^14^ or, more recently, optical fiber systems coupled to spectrum analyzers for real‐time dosimetry.^15^ The scanned image provides pixel values in the RGB color channels, representing the optical response of the film at each position.

The coloration of radiochromic film after irradiation is related to the absorbed dose at each point through a calibration function, typically a non‐linear relationship that depends on the film type and dose range. Common calibration models include polynomial, rational, exponential, and logistic functions,^16^ obtained by curve fitting between the optical density change and the corresponding absorbed dose measured with an absolute dosimeter, such as an ionization chamber. The resulting function allows the conversion of optical density into absorbed dose for films irradiated under conditions equivalent to those used for calibration.

Two major factors influence the generation of calibration curves and, consequently, the accuracy of dose measurements. The first concerns parameters that affect the optical density response of the film, such as film batch,^17^ ambient temperature,^18^ beam energy,^19^ scanning time,^20^ and mechanical handling.^21^ The second involves scanner‐related artifacts, including defective pixels, image noise from scratches or dust, lateral response artifact (LRA) due to scanner optics,^22^, ^23^ and orientation dependence with respect to the scanner lamp.^14^, ^24^ Several protocols have been proposed to mitigate these effects, notably the AAPM report,^25^ which summarizes the most effective irradiation and scanning procedures. Additionally, computational correction methods have been developed, such as multichannel analysis,^26^ lateral response modeling,^22^ filtering techniques,^16^, ^27^ recalibration strategies,^28^ and more recently, neural network‐based approaches.^29^ To address these well‐known film and scanner uncertainties, *FilmQADose* integrates multichannel dose reconstruction and image filtering options to improve dosimetric accuracy.

Various software tools have been developed to generate calibration curves, produce dose maps, and perform comparative analyses using different techniques. Commercially available solutions include FilmQA Pro (Ashland),^30^ myQA software (IBA Dosimetry),^31^ Radiochromic.com,^32^ Remote Dosimetry Services (RTsafe),^33^ RIT Film (Radiological Imaging Technology),^34^ and Verisoft (PTW Dosimetry).^35^ However, these commercial tools require paid licenses,^36^ restricting access to film dosimetry in facilities with limited resources. On the other hand, researchers developing novel software for film dosimetry use tools such as MATLAB,^37^ ImageJ,^38^ and R,^39^ sometimes restraining the accessibility of those research results and new methods to a niche of people who are familiar with such tools, not a generality in the clinical medical physicists community. Furthermore, there are a few recognized medical image analysis free software tools that provide partial support for film dosimetry, like the C++ based xgrid3d project,^40^ but none of them is specifically suited for the present necessity, nor are they easily modified to include new methods and techniques.

Consequently, the present work aimed to develop and evaluate an open‐source, user‐friendly software tool for radiochromic film dosimetry QA, incorporating calibration curve generation, single‐ and multichannel dose reconstruction, automated film‐to‐plan alignment, and gamma (Γ) comparison for quantitative analysis. The software and documentation are publicly available at our project webpage: https://eduardoh27.github.io/FilmQADose eduardoh27.github.io/FilmQADose.

## 2 ACQUISITION AND VALIDATION METHODS

### 2.1 General algorithm

**FIGURE 1:**
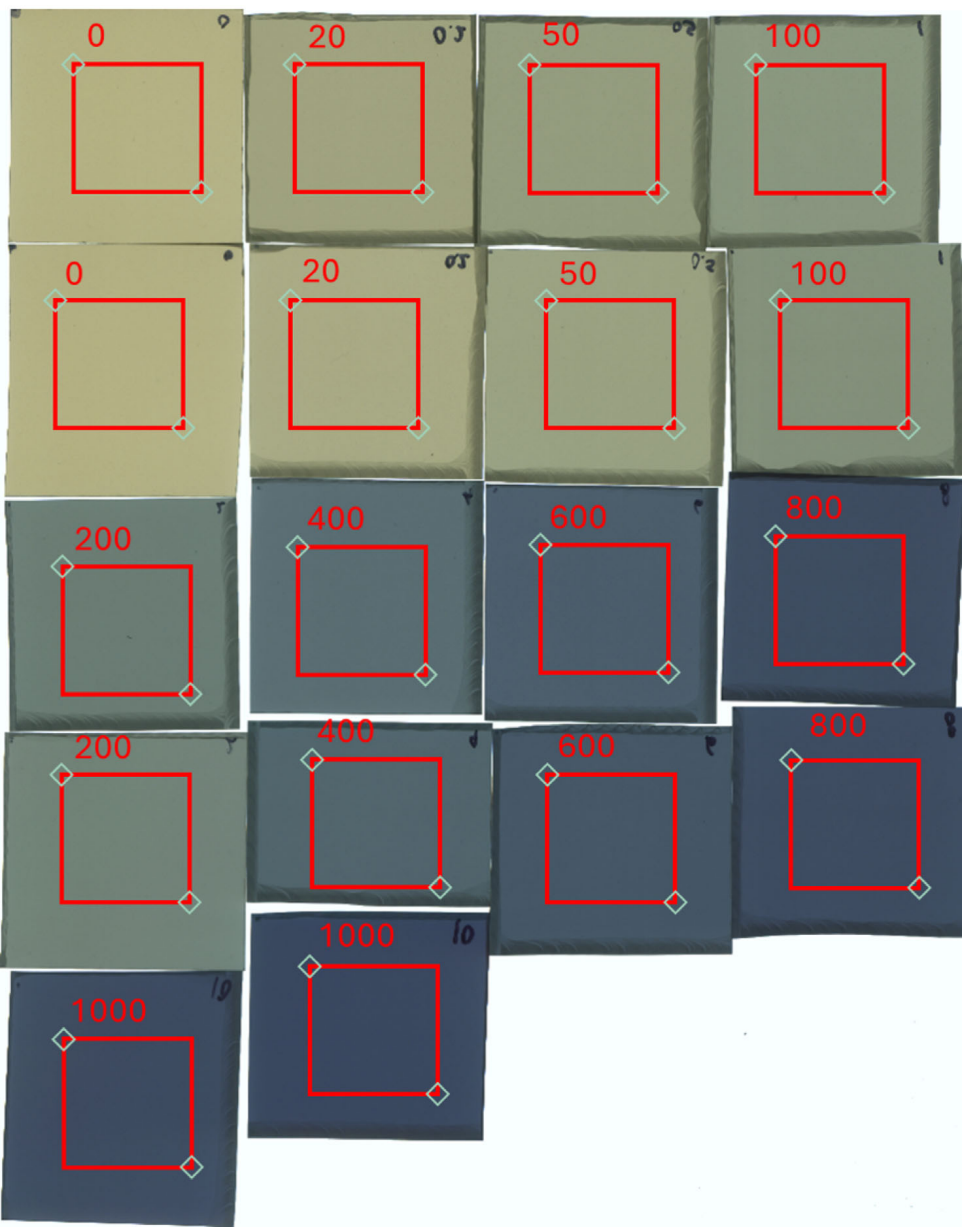
Screenshot of the FilmQADose software showing the ROI placement (red squares) on scanned Gafchromic EBT3 films irradiated with doses from 0 to 10 Gy.

Each pixel in an image contains color information in red, green and blue channels, represented by a number between 0 and $2^{16}-1$ in the 48‐bit color depth configuration. This number is associated with the intensity of measured color in the pixel. *Transmittance* (T) and *optical density* (OD) are defined for each pixel as:

(1) $$\begin{array}{cc} T & =\frac{PV}{2^{16}-1},\\ OD & =-\log_{10}T, \end{array}$$

where PV denotes the pixel information value in each channel.^27^ *FilmQADose* allows the user to extract average transmittance measurements from digitized images by selecting rectangular ROIs from which statistics are calculated, where PV statistics correspond to the mean pixel values within each ROI. Average transmittance in each channel was extracted by choosing a 2cm×2cm ROI on each film's center as shown in Figure 1.

Most models describing measured color dependence with absorbed dose are better suited to work with transmittance or optical density values. Some models relate absorbed dose with the more useful quantity of net change in film color, represented by:

(2) $$\begin{array}{cc} netT & =T_{f}-Ti,\\ netOD & =OD_{f}-OD_{i}, \end{array}$$

where $T_{i},OD_{i}$ and $T_{f},OD_{f}$ represent transmittance and optical density of the film before and after irradiation, respectively. Uncertainties are determined by the standard deviation of transmittance measurements over a set of pixels with equal dose.^27^ When netOD is used, the uncertainty is calculated as:

(3) $$\sigma_{{\mathrm{netOD}}^{i}}=\frac{1}{\ln(10)}\sqrt{{\left(\frac{\sigma_{PV_{f\;}}}{PV_{f\;}}\right)}^{2}+{\left(\frac{\sigma_{PV_{i\;}}}{PV_{i\;}}\right)}^{2}}.$$

If N film pieces are irradiated with the same dose to reduce variance, overall OD is calculated as the weighted average:

(4) $$\bar{\mathrm{net}OD}=\sum_{i=1}^{N}\left(\omega_{i}\cdot \mathrm{net}OD_{i}\right),$$

with weights given by:

(5) $$\omega_{i}=\frac{1/{\left(\sigma_{\mathrm{net}OD_{i}}\right)}^{2}}{\sum_{i=1}^{N}\left(1/{\left(\sigma_{{\mathrm{netOD}}_{i}}\right)}^{2}\right)},$$

and total uncertainty:

(6)

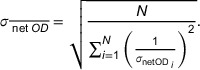




*FilmQADose* allows selection of an adequate model function between the most commonly used.^16^ In the present case, the manufacturer's suggested model for the EBT3 film, named a rational curve on transmittance values, was used:

(7) $$D=\frac{A\cdot netT}{1-B\cdot netT},$$

where A and B are the model parameters to be fitted and D is the corresponding absorbed dose. The fitting was performed over a dose range of 0–10 Gy, consistent with the calibration irradiations performed in this study. This function can be fitted to each color channel, with the red channel showing better prediction power due to the large film color variation in this part of the spectrum under radiation absorption.

Furthermore, this choice of calibration function is well suited for the multichannel method^26^ application, another efficient noise reduction technique that allows splitting of the dose‐dependent part of the film, associated to color change by dose absorption, from the dose‐independent part, associated with heterogeneities on the film or scanner defects. This method assumes that dose in each point is predicted with individual channel information ($D_{i}$) and can be calculated as:

(8) $$D_{i}=f_{i}\left(OD_{i}\cdot t\right),$$

where $f_{i}$ is the calibration function associated with the $i^{th}$ channel (R,G, and B) and t is the parameter separating the independent from the dependent dose part. To calculate the absolute dose, we find $t_{min}$, that minimizes the squared difference of dose individually predicted by each channel, that is, we find t satisfying the optimization problem given by:

(9) $$\min_{t}\sum_{i,j=R,G,B}{\left(D_{i}\left(t\right)-D_{j}\left(t\right)\right)}^{2}.$$

This problem is solved by using a standard Newton–Raphson algorithm with a maximum of 100 iterations. t values for each point in the image represent a map of separated non‐homogeneities in the original digitized film.

With the single‐channel method for calibration, dose maps are generated using the calibration function point by point on the digitized image. In the multichannel dosimetry method, dose in each point is predicted by use of the dose average predicted with each channel with equation 8, after solving the minimization problem. Generated maps are stored as DICOM 2D Map format.

For the sake of comparison, a polynomial calibration function was also used:

(10) $$D=A\cdot \left(netOD\right)+B\cdot {\left(netOD\right)}^{n},$$

where A, B, and n are the parameters to be fitted.

Traditionally, alignment between the digitized film map and the TPS‐generated dose map is assisted by fiducial markers printed on the film. These marks provide a visual guide to manually match both maps and ensure spatial correspondence. In addition to this manual approach, we implemented an automatic alignment method based on the *template matching* technique,^41^ a classic pattern recognition algorithm.

Template matching consists of identifying the location of a reference pattern (template) within a target image by measuring similarity at each location. In our case, the TPS map is treated as the template, while the digitized film is the target image. The method computes a *cross‐correlation* between both images:

(11) $$G\left[i,j\right]=\sum_{u=-k}^{k}\sum_{v=-k}^{k}H\left[u,v\right]F\left[i+u,j+v\right],$$

where H is the template image (TPS dose map), F is the film image, and G[i,j] is the correlation score at position [i,j] of the result matrix. The indices u,v iterate over the template dimensions (assumed to be square with half‐size k).

To account for variations in absolute intensity and control for relative brightness, normalization is performed.^42^ We use the normalized cross‐correlation (NCC), defined as:

(12) $$E_{\text{NCC}}\left(u\right)=\frac{\sum_{i}\left[I_{0}\left(x_{i}\right)-\bar{I_{0}}\right]\left[I_{1}\left(x_{i}+u\right)-\bar{I_{1}}\right]}{\sqrt{\sum_{i}{\left[I_{0}\left(x_{i}\right)-\bar{I_{0}}\right]}^{2}}\sqrt{\sum_{i}{\left[I_{1}\left(x_{i}+u\right)-\bar{I_{1}}\right]}^{2}}},$$

where $I_{0}$ and $I_{1}$ represent the pixel values of the template and target image, respectively. $\bar{I_{0}}$ and $\bar{I_{1}}$ are their corresponding mean intensities, computed as:

(13) $$\bar{I_{0}}=\frac{1}{N}\sum_{i}I_{0}\left(x_{i}\right)\;\text{and}\;\bar{I_{1}}=\frac{1}{N}\sum_{i}I_{1}\left(x_{i}+u\right).$$

The template‐matching algorithm first builds a correlation map between the film and the TPS template, and then identifies the peaks in this map as candidate match locations.

The Γ value is a standard metric used in clinical practice that allows differences between imparted and planned dose distributions to be quantified. To calculate the Γ value in each pixel, $\Gamma ({\vec{r}}_{c},{\vec{r}}_{m})$ is defined as:

(14) $$\Gamma \left({\vec{r}}_{c},{\vec{r}}_{m}\right)=\sqrt{\frac{|{\vec{r}}_{c}-{\vec{r}}_{m}{|}^{2}}{DTA^{2}}+\frac{|D\left({\vec{r}}_{c}\right)-D\left({\vec{r}}_{m}\right){|}^{2}}{DD^{2}\cdot D{\left({\vec{r}}_{c}\right)}^{2}}},$$

where ${\vec{r}}_{c}$ denotes pixel position in the calculated dose map measured in millimeters, $D({\vec{r}}_{c})$ the corresponding dose in such point and ${\vec{r}}_{m}$, $D({\vec{r}}_{m})$ denote the same quantities in the measured dose map. The absolute Γ matrix is calculated as the minimum of $\Gamma ({\vec{r}}_{c},{\vec{r}}_{m})$ over all measured points in the film, that is, $\Gamma \left({\vec{r}}_{c}\right)=\min_{m}\Gamma \left({\vec{r}}_{c},{\vec{r}}_{m}\right)$. Furthermore, DTA (distance to agreement) and DD (dose difference) parameters tune the comparison test desired accuracy, meaning the distance range and dose difference at which agreement of the two plans is considered.

### 2.2 Implementation

To facilitate source code modification and easiness to add new features to the film QA software, the Python programming language was chosen, as it is a simple yet powerful tool with open‐source libraries well suited for digital image handling and analysis. Furthermore, research code written in other languages, such as MATLAB and R, can be linked to *FilmQADose* through available language interfaces. The main libraries used by this software are: NumPy^43^ for array‐based operations, SciPy^44^ for curve fitting calculations using the Levenberg–Marquardt algorithm^45^, ^46^ and interpolation functions, Matplotlib^47^ for plotting and visualization purposes, tifffile^48^ for TIFF image reading and writing functionalities, Pydicom^49^ for DICOM file processing, PyMedPhys^50^ for Γ analysis evaluation, OpenCV^51^ for template matching algorithms, PyQtGraph^52^ for handling concurrent Regions of Interest (ROIs) and scikit‐image^53^ for image conditioning and re‐scaling. These functionalities are integrated into a graphical user interface (GUI) developed with the PySide6 framework,^54^ offering user‐friendly access to the analysis workflows.

All images are subjected to a filtering process for noise reduction produced by scanner digitization. A combination of a median filter for spike removal and an adaptive Wiener filter^55^ for noise reduction can be applied, which are available options in the scikit‐image Python library. However, new filters and noise reduction techniques can be easily implemented in the program source code.

In general, template matching requires a collection of templates that include several object examples, a range of scales at which the object may appear, and a variety of possible orientations. However, in our case, only a single template is needed: the TPS dose map. This is valid since the TPS map can be conceptualized as a smaller structure embedded within the digitized film. Scaling differences are not relevant, as both images (TPS and film) are rescaled to match their real‐world dimensions using metadata extracted from their DICOM and TIFF formats, respectively. Nevertheless, to account for a potential angular misalignment introduced during the experimental scanning setup, the TPS template is rotated by small increments ($\pm 1^{\circ }$, $\pm 2^{\circ }$, and $\pm 3^{\circ }$) to determine the orientation that maximizes the normalized cross‐correlation and ensures optimal alignment.

To ensure that both images are aligned before performing template matching, the digitized film is pre‐processed by applying a vertical reflection followed by a $180^{\circ }$ rotation. This pre‐processing step is specific to our scanner configuration, where the film orientation during scanning required such transformation to match the TPS coordinate system, and it can be customized for other setups.

Different dose normalization approaches can be selected during the quantitative analysis, including global normalization (using the maximum dose of the map) and local normalization (relative to each point's planned dose),^56^ as well as absolute doses (no normalization). For the present work, global normalization was used to facilitate comparison of the spatial dose distributions by omitting slight absolute dose differences between maps. Quantitative comparison analysis was performed by calculating the Γ matrix between the determined dose map and the original TPS planned map.

### 2.3 Validation

**FIGURE 2:**
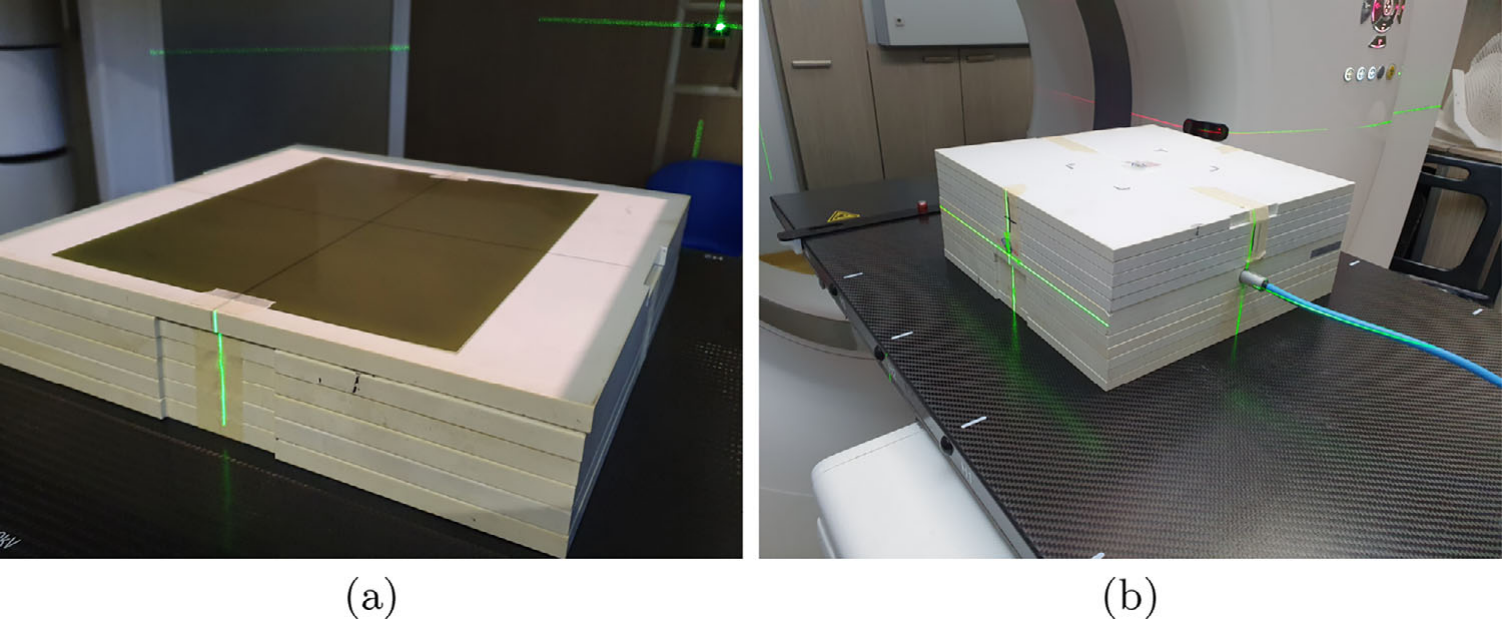
Experimental setups for dose verification. (a) Full uncut film on 5 cm of solid water and alignment with isocenter. (b) Ionization chamber setup for independent dose measurements.

**FIGURE 3:**
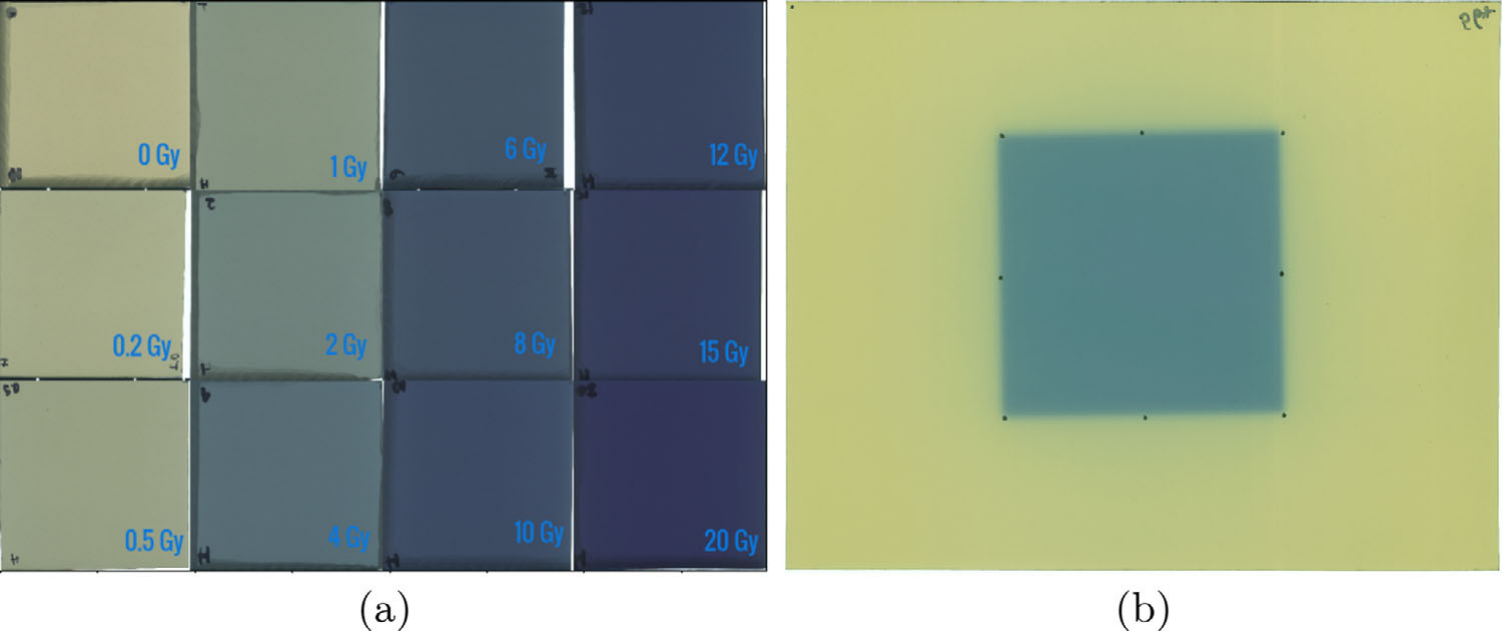
Calibration and verification radiochromic films. (a) Gafchromic EBT3 films irradiated with reference doses ranging from 0 to 20 Gy, used to construct the calibration curve. (b) Film irradiated with a uniform 5 Gy square field for dose distribution verification.

To establish a calibration curve, 5cm×5cm film pieces were cut from an $8^{\prime \prime }\times 10^{\prime \prime }$ Gafchromic EBT3 film (batch number: 12121701) using regular scissors. To ensure that each film was aligned with the original orientation of the $8^{\prime \prime }\times 10^{\prime \prime }$ film, marks were added at its right‐bottom corner. 5 cm solid water blocks were placed under and on top of the film to account for backscatter radiation and to reach electronic equilibrium, respectively. Each film was aligned with accelerator isocenter and a 10cm×10cm field was employed to ensure homogeneity. Irradiation was performed independently to avoid scattered radiation effects on the absorbed dose measurement (Figure 2a). Twelve dose values from 0 to 20 Gy (where the EBT3 film started to saturate) were imparted on three film sets to reduce final calibration variance, reaching a total of 36 irradiated films. Each dose was independently measured under the same conditions using an ionization chamber, as shown in Figure 2b. The resulting film pieces are shown in Figure 3a.

For dose verification purposes in a large homogeneous field, a 5 Gy, 10cm×10cm field was irradiated on a full film, shown in Figure 3b. Applying the same methodology, a pyramidal plan and breast IMRT plan were irradiated, whose 2D dose maps at isocenter were calculated by Eclipse 15.5 TPS^57^ (Varian Medical Systems). These plans were imported as DICOM RT Dose files with a resolution of 256×256 pixels, without any resampling. Fiducial point marks were printed on each film to facilitate later alignment with the calculated dose map. Following the AAPM TG‐235 recommendations,^25^ the films were scanned 48 h after irradiation. Although TG‐235 reports that measurements performed after 24 h show minimal variation, a 48‐h waiting period was adopted in our clinical routine to further minimize residual variations. Films were scanned using a previously cleaned Microtek ScanMaker 1000XL device, with 16‐line sampling, 48‐bit color depth, 100 dpi resolution. All automatic color corrections, sharpening, and filtering features were fully disabled to ensure unprocessed raw image data for analysis. Care must be taken to avoid film rotation on the scanner bed, as it may affect optimal density measurement due to polarized light effects.^58^ To avoid device temperature effects on film coloration, five empty test scans were performed, aiming to reach a stable temperature before data acquisition. To reduce lateral response effect induced by the scanner, small film pieces were scanned individually in the same scanner position each time. The resulting images were RGB matrices saved as uncompressed TIFF files.

## 3 RESULTS

**FIGURE 4:**
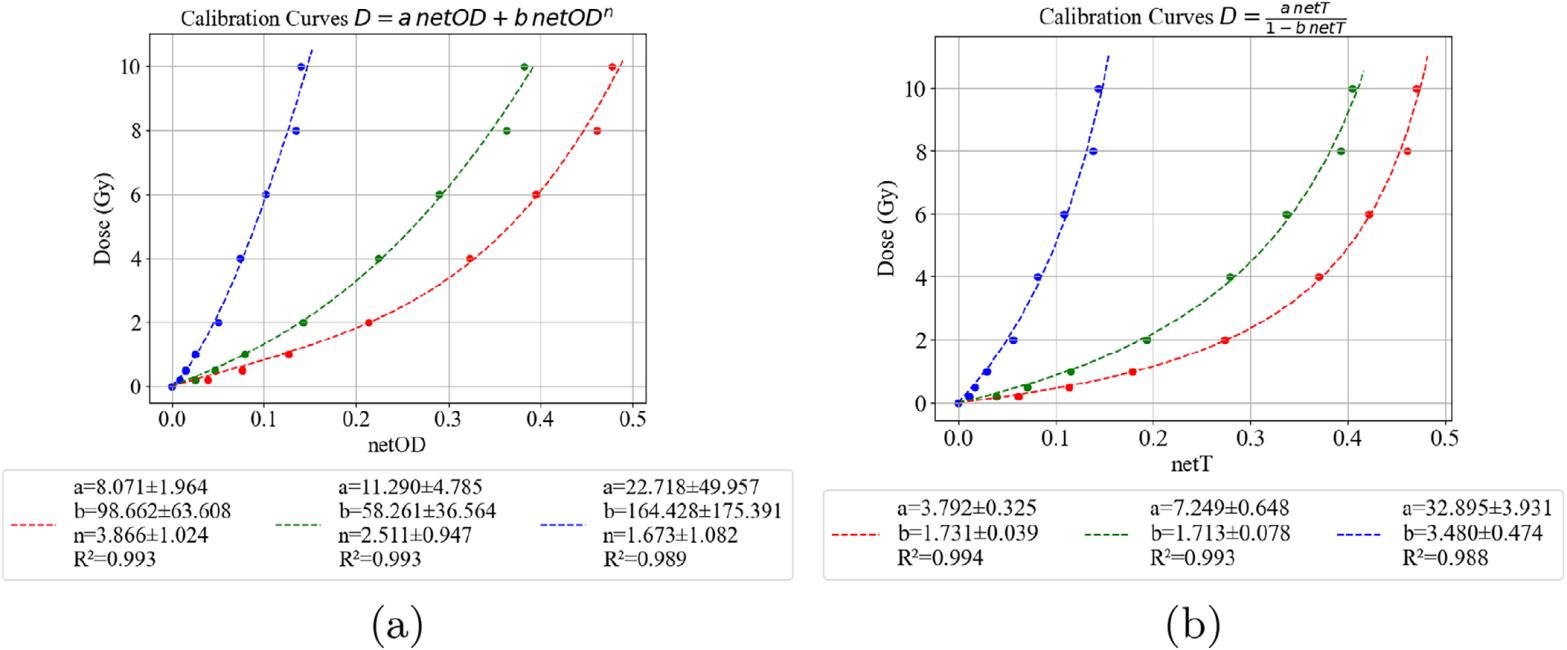
Comparison of calibration models using the single‐channel approach. (a) Polynomial calibration curves based on net optical density (netOD) for the red, green, and blue channels. (b) Rational calibration curves based on net transmittance (netT) for the same RGB channels. Each model was fitted using nine dose levels in the range of 0 to 10 Gy, with respective calibration parameters and goodness‐of‐fit values shown below.

Figures 4a and 4b show the calibration function obtained with the data points in the range from 0 to 10 Gy using polynomial and rational functions, respectively. Modeling with a rational function of transmittance‐absorbed dose relation fits better in the worked dose range, as suggested by the film manufacturer. Error bars, computed as previously described,^27^ are smaller than mark points and are hardly visible. Therefore, taking the dose range used in the irradiated treatment plans into account, the rational calibration curve with data from 0 to 10 Gy to convert transmittance information into 2D dose maps was used.

**FIGURE 5:**
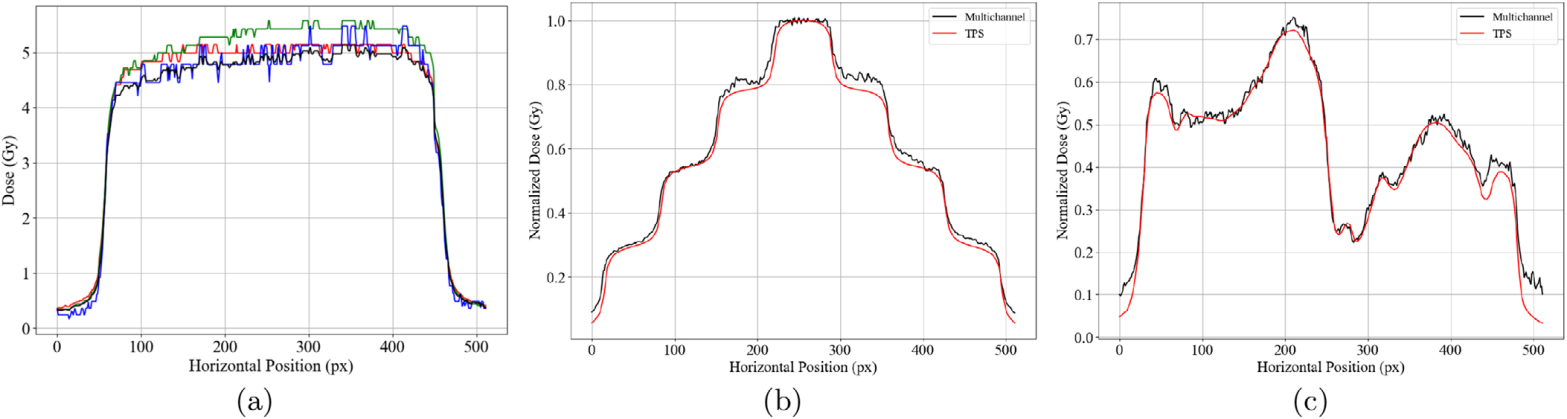
Dose profile comparison. (a) Horizontal central axis dose profiles for the 5 Gy square irradiation, showing single‐channel results for red, green, and blue (RGB) channels, as well as the multichannel profile (black). (b) Normalized dose profile for a pyramidal irradiation plan, comparing TPS (red) and film measurement (black). (c) Normalized dose profile for a breast cancer treatment plan, comparing TPS (red) and film measurement (black).

Figure 5a shows a horizontal profile of the 5 Gy, 10cm×10cm field dose map obtained by the single‐channel and multichannel methods with the polynomial calibration curve. High noise using the blue channel is evidenced. The red channel provides an accurate dose measurement in the central region, whereas the green channel exhibits an overestimation. The multichannel method shows its noise reduction property, but also a light underestimation of dose as opposed to the single‐channel method, as obtained in previous studies.^26^

**FIGURE 6:**
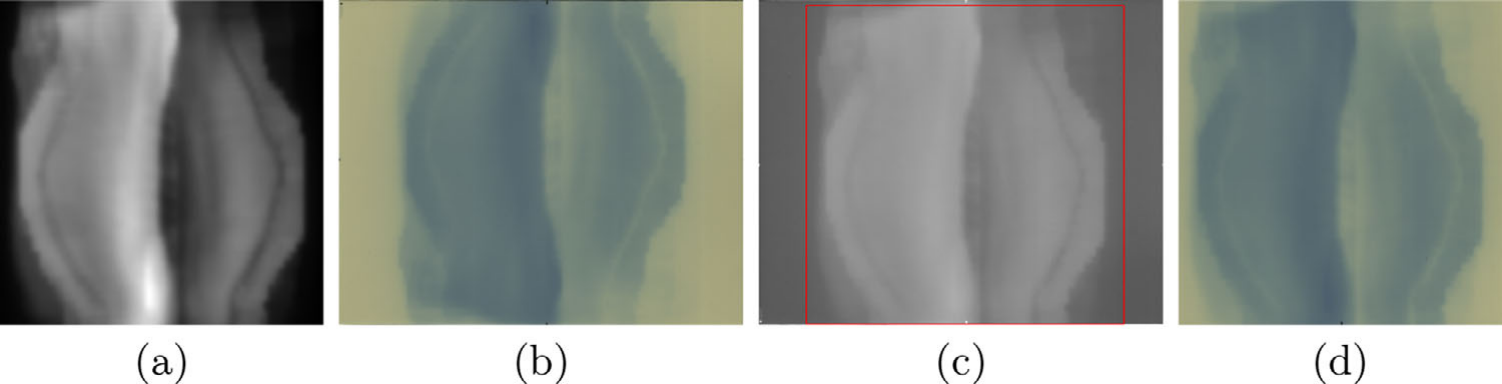
Template matching for alignment in breast IMRT verification. (a) Planned dose distribution from the treatment planning system (TPS). (b) Digitized film after irradiation. (c) Result of the template matching algorithm, highlighting the detected region in red. (d) Cropped sub‐image corresponding to the matched region extracted from the film.

The irradiated film for the breast IMRT plan and its corresponding TPS map are shown in Figure 6a,b, respectively, while Figure 5b,c show normalized dose profiles obtained along the horizontal axis of both plans. Agreement was found between predicted and measured maps, with the maximum dose difference less than 5% of the predicted dose. However, noise is visible in some regions of the film dose map. This could not be fully eliminated, likely due to hardware limitations of the scanner, which had areas of reduced pixel sensitivity and some non‐functional pixels. This was the minimum noise level achievable with the available device, and it may be improved with more advanced scanning systems.

**FIGURE 7:**
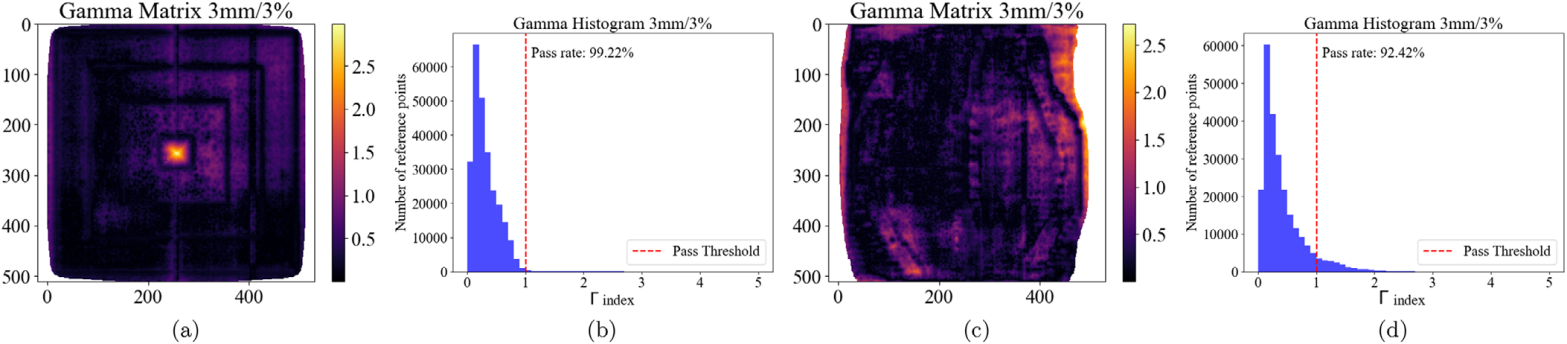
Gamma index analysis using the 3%/3 mm criterion for two radiotherapy treatment plans. (a) Gamma index matrix for the pyramidal plan, showing spatial agreement between the measured and planned dose distributions. (b) Gamma histogram for the pyramidal plan, with a pass rate of 99.22%. (c) Gamma index matrix for the breast treatment plan. (d) Gamma histogram for the breast plan, showing a pass rate of 92.42%.

Finally, Figure 7 shows the corresponding Γ matrices for each plan, and histograms that represent the Γ distribution among all tested pixels. For the pyramidal plan, Γ analysis was performed by using 3 mm DTA, 3% DD and using a dose threshold of 5% of the maximum dose. Good correspondence between the measured and planned dose distributions was achieved, having 99.22% of the tested pixels passing the criteria. Γ analysis of the IMRT plan was performed using 3%/3 mm parameters, resulting in 92.42% of evaluated pixels passing the test. Discrepancy is due to higher plan complexity and the difficulty of manually superposing both maps.

## 4 DISCUSSION

The calibration results support selecting a rational transmittance–dose model for dose reconstruction within 0–10 Gy. This choice aligns with manufacturer guidance.

Single‐channel behavior demonstrates practical processing choices for film QA. The elevated noise in the blue channel elevated contrasts with the more stable red/green channels in the field center, indicating that reliance on red/green channels is preferable when using single‐channel methods. Furthermore, in the absence of LRA correction, the green channel is generally preferred for two‐dimensional film dosimetry, as it exhibits lower susceptibility to lateral response artifacts and improved gamma agreement across the scan field compared with the red and blue channels.^22^ The multichannel approach effectively suppresses noise but introduces a small underestimation relative to single‐channel processing, as previously reported.^26^ In QA workflows, this trade‐off means multichannel processing can yield smoother profiles, whereas single‐channel reconstructions may recover slightly higher doses.

Agreement between the breast IMRT film and TPS, with maximum profile deviations <5%, indicates that, under the present calibration and processing, film‐based verification reproduces planned dose trends for this case. The residual map noise is attributable to documented scanner limitations (pixel non‐uniformity and isolated failures), which set a floor on achievable smoothness independent of the calibration model. Consequently, device uniformity and detector integrity are limiting factors for further noise reduction in the current setup.

The Γ analysis values are consistent with plan characteristics and registration conditions. The pyramidal plan's 99.22% passing rate (3%/3 mm, 5% threshold) reflects its simpler modulation, whereas the IMRT plan's 92.42% passing rate at 3%/3 mm is compatible with greater modulation complexity and with registration uncertainty from manual alignment. These observations indicate that discrepancies are dominated by plan complexity and registration, rather than by the selected calibration function. Within the presented data, the most direct ways to improve agreement are to minimize registration uncertainty (for example by reducing or avoiding manual superposition) and to address scanner non‐uniformity. Despite the difficulties mentioned, *FilmQADose* allows users to perform appropriate film QA dosimetry by calibration curve generation, dose map and dose profile visualization and planned‐to‐measured dose Γ analysis.

The applicable dose range for the current implementation is 0.5–20 Gy, and the supported field size range extends from 5cm×5cm to 22cm×22cm. These limits correspond to the calibration conditions and film scanner configuration used in this study and define the range over which accurate reconstruction and comparison can be expected.

Nevertheless, the current version of *FilmQADose* presents several limitations that will guide future development. The implemented multichannel calibration does not yet incorporate state‐of‐the‐art recalibration or machine‐learning–based correction methods, which could further improve dose accuracy. Additionally, LRA correction is not yet implemented, which may explain the observed asymmetry in Figure 5a. Another potential source of uncertainty is the aging flatbed scanner used in this study, whose non‐uniform response and reduced sensitivity likely contributed to residual noise.

As an open‐source initiative, *FilmQADose* is designed to evolve through continuous refinement and collaborative contributions from the medical physics community. Future releases will aim to expand functionality, enhance robustness, and integrate correction methods to ensure reliability and clinical applicability.

## 5 CONCLUSION

*FilmQADose* is a free, open‐source, Python‐based tool that delivers an end‐to‐end workflow for radiochromic film QA, including calibration, single‐ and multichannel dose reconstruction, automated film‐to‐plan alignment, and DICOM‐RT Γ‐comparison for quantitative evaluation.

In the evaluated cases (pyramidal and breast IMRT), the software reproduced planned dose trends and yielded Γ pass rates of 99.22% for the pyramidal plan and 92.42% for the breast IMRT plan using a global 3%/3 mm criterion with a 5% threshold. Consistent with clinical QA practice, the former indicates high agreement, whereas the breast result is acceptable and emphasizes opportunities for improvement. The current implementation is applicable in the dose range 0.5–20 Gy and field sizes 5cm×5cm to 22cm×22cm, aligned with the calibration and scanning conditions used in this study.

Limitations and uncertainty sources remain. The observed profile asymmetry cannot be uniquely attributed to the beam, film, or scanner. Contributing factors may include the absence of an explicit LRA correction, non‐uniformity in a legacy scanner (including dust, scratches, and defective sensor pixels), and potential mechanical stress at film cut edges that alters layer structure. As an openly available project, *FilmQADose* is intended to evolve through iterative enhancements that expand functionality and reliability while maintaining reproducible workflows for film‐based QA in radiotherapy.

## AUTHOR CONTRIBUTIONS

Concept and design: Juliana Sandoval‐Navia, Jaider Vásquez. Data collection: Juliana Sandoval‐Navia, Jaider Vásquez, Carlos Contreras‐Quiroz. Software development: Eduardo Herrera‐Alba, Carlos Contreras‐Quiroz. Data analysis and validation: Eduardo Herrera‐Alba, Carlos Contreras‐Quiroz, Carlos Ávila, Juliana Sandoval‐Navia, Jaider Vásquez. Manuscript preparation: Eduardo Herrera, Carlos Contreras‐Quiroz, Juliana Sandoval‐Navia, Carlos Ávila, Jaider Vásquez. Research management and supervision: Carlos Ávila, Juliana Sandoval‐Navia.

## 6 CONFLICTS OF INTEREST STATEMENT

The authors have no relevant conflicts of interest to disclose.

## Data Availability

The data and code that support the findings of this study are openly available at: eduardoh27.github.io/FilmQADose.

## References

[1] Hassani H , Nedaie HA , Zahmatkesh MH , Shirani K . A dosimetric study of small photon fields using polymer gel and Gafchromic EBT films. Med Dosim. 2014;39(1):102–107. 24388694 10.1016/j.meddos.2013.10.007

[2] Asgharizadeh S , Bekerat H , Syme A , et al. Radiochromic film–based quality assurance for CT‐based high‐dose‐rate brachytherapy. Brachytherapy. 2015;14(4):578–585. 25865477 10.1016/j.brachy.2015.02.192

[3] Schumer W , Fernando W , Carolan M , et al. Verification of brachytherapy dosimetry with radiochromic film. Med Dosim. 1999;24(3):197–203. 10555059 10.1016/s0958-3947(99)00018-7

[4] Massillon‐JL G , Chiu‐Tsao ST , Domingo‐Munoz I , Chan MF . Energy dependence of the new Gafchromic EBT3 Film: dose response curves for 50 KV, 6 and 15 MV X‐ray beams. Int J Med Phys Clin Eng Radiat Oncol. 2012;01(02):60–65. 10.1118/1.4735153 PMC13387173 28517140

[5] Cheung T , Butson MJ , Yu PK . Independence of calibration curves for EBT Gafchromic films of the size of high‐energy X‐ray fields. Appl Radiat Isot. 2006;64(9):1027–1030. 16774834 10.1016/j.apradiso.2006.04.006

[6] Darafsheh A , Ghaznavi H . A review on radiochromic film dosimetry in radiation therapy. J Appl Clin Med Phys. 2025;26(12):e70365. 41330745 10.1002/acm2.70365 PMC12672139

[7] Casolaro P . Radiochromic films for the two‐dimensional dose distribution assessment. Appl Sci. 2021;11(5):2132.

[8] Williams M , Metcalfe P . Radiochromic Film Dosimetry and its Applications in Radiotherapy. AIP Conference Proceedings. 2011;1345(1):75–99.

[9] Das IJ . Radiochromic Film: Role and Applications in Radiation Dosimetry. 1st ed. CRC Press; 2017.

[10] Ashland, Bridgewater, NJ, USA. Accessed June 19, 2025, http://www.gafchromic.com/gafchromic‐film/radiotherapy‐films/EBT/index.asp

[11] Gafchromic . User Manual. Accessed June 19, 2025, http://www.gafchromic.com/documents/EBT3_Specifications.pdf

[12] Gafchromic . User Manual. Accessed June 19, 2025, http://www.gafchromic.com/documents/EBTXD_Specifications_Final.pdf

[13] Palmer AL , Nash D . Radiochromic film dosimetry in radiotherapy: a survey of current practice in the United Kingdom. Br J Radiol. 2024;97(1155):646–651. 38273671 10.1093/bjr/tqae008 PMC11027307

[14] Butson MJ , Yu PK , Cheung T , Metcalfe P . Radiochromic film for medical radiation dosimetry. Mater Sci Eng R Rep. 2003;41(3‐5):61–120.

[15] Casolaro P , Campajola L , Breglio G , et al. Real‐time dosimetry with radiochromic films. Sci Rep. 2019;9(1):5307. 30926839 10.1038/s41598-019-41705-0 PMC6440967

[16] Santos T , Ventura T , Carmo Lopes M . A review on radiochromic film dosimetry for dose verification in high energy photon beams. Radiat Phys Chem. 2021;179:109217.

[17] Kairn T , Aland T , Kenny J . Local heterogeneities in early batches of EBT2 film: a suggested solution. Phys Med Biol. 2010;55(15):L37–L42. 20616403 10.1088/0031-9155/55/15/L02

[18] Rink A , Lewis DF , Varma S , Vitkin IA , Jaffray DA . Temperature and hydration effects on absorbance spectra and radiation sensitivity of a radiochromic medium. Med Phys. 2008;35(10):4545–4555. 18975701 10.1118/1.2975483 PMC2736758

[19] Bassi S , Cummins D , McCavana P . Energy and dose dependence of GafChromic EBT3‐V3 film across a wide energy range. Rep Pract Oncol Radiother. 2020;25(1):60–63. 31889923 10.1016/j.rpor.2019.12.007 PMC6931201

[20] Reinstein LE , Gluckman GR , Meek AG . A rapid colour stabilization technique for radiochromic film dosimetry. Phys Med Biol. 1998;43(10):2703–2708. 9814510 10.1088/0031-9155/43/10/001

[21] Yu PKN , Butson M , Cheung T . Does mechanical pressure on radiochromic film affect optical absorption and dosimetry?. Australas Phys Eng Sci Med. 2006;29(3):285–287. 17058593 10.1007/BF03178580

[22] Lewis D , Chan MF . Correcting lateral response artifacts from flatbed scanners for radiochromic film dosimetry. Med Phys. 2014;42(1):416–429. 10.1118/1.4903758 PMC5148133 25563282

[23] Palmer AL , Dimitriadis A , Nisbet A , Clark CH . Evaluation of Gafchromic EBT‐XD film, with comparison to EBT3 film, and application in high dose radiotherapy verification. Phys Med Biol. 2015;60(22):8741. 26512917 10.1088/0031-9155/60/22/8741

[24] Darafsheh A . On energy dependency, spectral properties, and orientation dependency of EBT3, EBT‐XD, MD‐V3, and HD‐V2 radiochromic films. Phys Med Biol. 2025;70(8):085015. 10.1088/1361-6560/adcafc 40203868

[25] Niroomand‐Rad A , Chiu‐Tsao ST , Grams MP , et al. Report of AAPM task group 235 radiochromic film dosimetry: an update to TG‐55. Med Phys. 2020;47(12):5986–6025. 32990328 10.1002/mp.14497

[26] Micke A , Lewis DF , Yu X . Multichannel film dosimetry with nonuniformity correction. Med Phys. 2011;38(5):2523–2534. 21776787 10.1118/1.3576105

[27] Devic S , Tomic N , Lewis D . Reference radiochromic film dosimetry: Review of technical aspects. Phys Med. 2016;32(4):541–556. 27020097 10.1016/j.ejmp.2016.02.008

[28] Ruiz‐Morales C , Vera‐Sánchez JA , González‐López A . Optimizing the recalibration process in radiochromic film dosimetry. Phys Med Biol. 2020;65(1):015016. 31746787 10.1088/1361-6560/ab58dd

[29] Chang L , Chen PY , Yeh SA , Kang CL , Su CT , Lee TF . Adaptive calibration of Gafchromic EBT3 film using generalized additive neural networks. Sci Rep. 2025;15(1):8208. 40065026 10.1038/s41598-025-92568-7 PMC11894168

[30] FilmQA Pro . Accessed June 19, 2025, http://www.gafchromic.com/filmqa‐software/filmqapro/index.asp

[31] IBA‐Dosimetry . Accessed June 19, 2025, https://www.iba‐dosimetry.com

[32] Radiochromic.com . Accessed June 19, 2025, https://radiochromic.com

[33] RTSafe . Accessed June 19, 2025, https://rt‐safe.com

[34] Radiological Imaging Technology . Accessed June 19, 2025, https://radimage.com/products/rit‐family‐of‐products/rit‐film/

[35] PTW Dosimetry . Accessed June 19, 2025, https://www.ptwdosimetry.com/en/products/verisoft

[36] Buddhavarapu A . A comparison of three‐film analysis software for stereotactic radiotherapy patient‐specific quality assurance. J Appl Clin Med Phys. 2024;25(3):e14203. 37937814 10.1002/acm2.14203 PMC10929995

[37] The MathWorks Inc . MATLAB version: 9.13.0 (R2022b). https://www.mathworks.com

[38] Rueden CT , Schindelin J , Hiner MC , et al. ImageJ2: ImageJ for the next generation of scientific image data. BMC Bioinformatics. 2017;18(1):529. 29187165 10.1186/s12859-017-1934-z PMC5708080

[39] R Core Team . R: A language and environment for statistical computing. https://www.R‐project.org

[40] Bolaños‐Puchet S . xgrid3d: A free software tool for quantitative image analysis and internal dosimetry. AIP Conf Proc. 2019;2090(1):030003.

[41] Gonzalez RC , Woods RE . Digital Image Processing. 4th ed. Pearson; 2018.

[42] Szeliski R . Computer Vision: Algorithms and Applications. Springer; 2022.

[43] Harris CR , Millman KJ , Walt SJ , et al. Array programming with NumPy. Nature. 2020;585(7825):357–362. 32939066 10.1038/s41586-020-2649-2 PMC7759461

[44] Virtanen P , Gommers R , Oliphant TE , et al. SciPy 1.0: fundamental algorithms for scientific computing in Python. Nat Methods. 2020;17:261–272. 32015543 10.1038/s41592-019-0686-2 PMC7056644

[45] Levenberg K . A method for the solution of certain non‐linear problems in least squares. Q Appl Math. 1944;2(2):164–168.

[46] Marquardt DW . An algorithm for least‐squares estimation of nonlinear parameters. J Soc Ind Appl Math. 1963;11(2):431–441.

[47] Hunter JD . Matplotlib: A 2D graphics environment. Comput Sci Eng. 2007;9(3):90–95.

[48] Gohlke C . cgohlke/tifffile: v2025.3.30. 2025. 10.5281/zenodo.15107527

[49] Mason D . pydicom/pydicom: pydicom 3.0.1. 2024. 10.5281/zenodo.13824606

[50] Biggs S , Jennings M , Swerdloff S , et al. PyMedPhys: A community effort to develop an open, Python‐based standard library for medical physics applications. J Open Source Softw. 2022;7(78):4555.

[51] Bradski G . The OpenCV Library. Dr Dobb's Journal of Software Tools. 2000.

[52] Luke Campagnola . Accessed June 19, 2025, http://www.pyqtgraph.org

[53] Walt S , Schönberger JL , Nunez‐Iglesias J , et al. scikit‐image: image processing in Python. PeerJ. 2014;2:e453. 25024921 10.7717/peerj.453 PMC4081273

[54] The Qt Company . Accessed June 19, 2025, https://doc.qt.io/qtforpython‐6

[55] Jin F , Fieguth P , Winger L , Jernigan E . Adaptive Wiener filtering of noisy images and image sequences. In: Proceedings of the 2003 International Conference on Image Processing . Vol 3. IEEE; 2003:349‐352.

[56] Miften M , Olch A , Mihailidis D , et al. Tolerance limits and methodologies for IMRT measurement‐based verification QA: Recommendations of Task Group No. 218. Med Phys. 2018;45(4):e53–e83. 29443390 10.1002/mp.12810

[57] Varian . Accessed June 19, 2025, https://www.varian.com/products/radiotherapy/treatment‐planning/eclipse

[58] Darafsheh A . Polarization properties of EBT3, EBT4, EBT‐XD, MD‐V3, and HD‐V2 radiochromic films. Phys Med Biol. 2025;70(20):20NT01. 10.1088/1361-6560/ae0bed 41059809

